# Equity in health financing of Guangxi after China’s universal health coverage: evidence based on health expenditure comparison in rural Guangxi Zhuang autonomous region from 2009 to 2013

**DOI:** 10.1186/s12939-017-0669-9

**Published:** 2017-09-29

**Authors:** Xianjing Qin, Hongye Luo, Jun Feng, Yanning Li, Bo Wei, Qiming Feng

**Affiliations:** School of Information And Management, Guangxi Medical University, 22 Shuangyong Road, Qingxiu District, Nanning, Guangxi Zhuang Autonomous Region China

**Keywords:** Health financing equity, Rural China, Expenditure, Catastrophic payments, Regressive

## Abstract

**Background:**

Healthcare financing should be equitable. Fairness in financial contribution and protection against financial risk is based on the notion that every household should pay a fair share. Health policy makers have long been concerned with protecting people from the possibility that ill health will lead to catastrophic financial payments and subsequent impoverishment. A number of studies on health care financing equity have been conducted in some provinces of China, but in Guangxi, we found such observation is not enough. What is the situation in Guagnxi? A research on rural areas of Guangxi can add knowledge in this field and help improve the equity and efficiency of health financing, particularly in low-income citizens in rural countries, is a major concern in China’s medical sector reform.

**Methods:**

Socio-economic characteristics and healthcare payment data were obtained from two rounds of household surveys conducted in 2009 (4634 respondents) and 2013 (3951 respondents). The contributions of funding sources were determined and a progressivity analysis of government healthcare subsidies was performed. Household consumption expenditure and total healthcare payments were calculated and incidence and intensity of catastrophic health payments were measured. Summary indices (concentration index, Kakwani index and Gini coefficient) were obtained for the sources of healthcare financing: indirect taxes, out of pocket payments, and social insurance contributions.

**Results:**

The overall health-care financing system was regressive. In 2013, the Kakwani index was 0.0013, the vertical effect of all the three funding sources was 0.0001, and some values exceeded 100%, indicating that vertical inequity had a large influence on causing total health financing inequity. The headcount of catastrophic health payment declined sharply between 2009 and 2013, using total expenditure (from 7.3% to 1.2%) or non-food expenditure (from 26.1% to 7.5%) as the indicator of household capacity to pay.

**Conclusion:**

Our study demonstrates an inequitable distribution of government healthcare subsidies in China from 2009 to 2013, and the inequity was reduced, especially in rural areas. Future healthcare reforms in China should not only focus on expanding the coverage, but also on improving the equity of distribution of healthcare benefits.

## Background

Health systems deliver preventive and curative health services that can make a substantial difference to peoples’ health. Healthcare financing should be equitable [1]. Thus, the fairness of health financing has become a major concern for both governments and its citizens. What constitutes a fair share depends on people’s expectations as to how health systems are financed [2, 3]. Fairness in financial contributions towards healthcare is a key component of modern day approaches to health system assessments [4]. Vertical equity means that people with dissimilar abilities should make dissimilar levels of contribution to the health-care financing system [5, 6]. By measuring progressivity, vertical and horizon equity can be analyzed [7, 8]. The means of financing health care has been identified as a barrier to access to healthcare and increases the likelihood of impoverishment of households [9, 10].

Nevertheless, in all countries, fairness in financial contribution embraces two critical aspects; that of risk pooling between the healthy and the sick, and risk sharing across wealth and income levels [11]. Regardless of income or wealth, risk sharing refers to the premise that equity does not mean equal contributions from all, but that contributions are greater from those who have more financial resources. However, citizens do not usually pay too much attention on the figures or meanings hidden in government reports, instead, they focus on what they can benefit from the policy, such as the proportion of the subsidies and whether they will fall into impoverishment after paying a medical bill.

The importance of health for all human lives means that concerns about its allotment are important to us all [2] and the distribution of health funding reflects the efficiency of a government. This is more so in developing countries such as China where direct payments (out-of-pocket payments) form a greater proportion of the sources of health-care financing [12–14].

China is a developing country with a massive population. In order to provide equitable access to healthcare for all, changes to health-care financing systems are being implemented with the aim of attaining universal coverage [15]. Guangxi is an autonomous region in western China and is one of the less developed regions of China which has aroused concern from both the government and local residents. In 2003, China adopted a new health insurance system, the New Cooperative Medical Scheme (NCMS), in rural areas where 80% of people were without health insurance of any kind [16]. While the rapid increase of health insurance coverage in rural China is certainly striking, it means little if the programs effectiveness is limited.

Researchers have conducted some studies on NCMS from different points of view. In the case of Fengshang county (a county located in Guizhou province, a developing western area next to Guangxi), Wang [17] demonstrated that in the NCMS pattern of “Low premium and High co-payment ratio”,the health financing inequity existed. Song [18] put forward that the utilization of Gini coefficient can measure and assess the impact of NCMS on population income equity, Ren [19] used the data of 3 pilot counties, pointed that low income groups with high health care need benefit little in easing financial burden from the NCMS programe. In Li’s study [20] in Heilongjiang province,she concluded that the health financing equity situation of NCMS non-participants were better than participants. While in Lu’s research [21], he argued that NCMS has promoted the equity in health care financing.

Currently, a limited number of studies investigating equity analysis have been conducted in Guangxi, particularly in rural areas. According to the China National Health Accounts Report [22], Guangxi has a heavy burden on medical expense in 2008, and the portion of out of pocket health expenditure as total health expenditure increased year by year, and it indicated the exist of health financial inequity. So what is the level of equity in health financing after universal health coverage in Guangxi compared with other districts? Is the situation in rural areas different from other observations? Based on the previous studies, it is reasonable to hypothesize that increased coverage may not result in better utilization of medical care and less impoverishment. Therefore, there is need to conduct a study in Guangxi to evaluate the health care financing equity after NCMS implemented, to figure out whether the NCMS relieve peasants of financial burden of health care by reducing out-of-pocket expenditures and narrow the gap between the wealthy and poor.

Financing of the NCMS for the central and western provinces of China differs from that eastern provinces according to economic levels. Since local governments have the authority to design and implement their own NCMS programs, the effects of the NCMS program might be heterogeneous. A new governmental public health service funding for rural areas was started in 2009. However, the funding for economically developing rural areas is still not so sufficient as that in economically developed rural areas [23]. Compared to developed provinces in China, Guangxi has more public health problems Low government health expenditures and inadequate health resources is a common problem in these developing regions [24]. Most poor people in Guangxi are villagers in remote and mountainous regions with low levels of education and weak awareness of health protection, all of which are susceptible risks of the spread of infectious diseases [25]. Even though the NCMS has witnessed a rapid expansion in coverage since its inception, people has lots of complaints in formal or informal surveys, and the arguments on the two Chinese phrases meaning "proper health care is difficult to get" and “proper health care is expensive” have gained currency in these years, reflecting ground realities.

This study was conducted in rural Guangxi by accessing the progressivity of health system and comparing changes in poverty conditions from 2009 to 2013. Results from this study can hopefully provide potential advice to health policy makers by addressing health service needs among those living in rural areas.

## Methods

### Study setting

This study was conducted in Guangxi province, an autonomous region located in western China with an area of 236,700 km^2^. The Han Chinese are the largest ethnic group, however over 14 million Zhuang, the largest minority ethnicity of China, live in Guangxi. Based on the Aronson–Johnson–Lampert approach, the distributive effect was split into its three components: progressivity, horizontal equity, and reranking [5]. We combine evidence from disaggregated measures (concentration curve and Lorenz curve) and summary indices (Gini coefficient, Kakwani index, and concentration index) to demonstrate the healthcare financing status and evaluate the effects of NCMS in rural Guangxi. And a comparison of impoverishment due to health expenditure is also done by calculating the incidence and intensity of catastrophic healthcare expenditure.

### Sampling

Rongxian and Luchuan counties were chosen as the sample counties in our project. The economic development and health services of the two counties are on the same level, and in the middle economic level of Guangxi. Rongxian and Luchuan counties are both located in the southeastern part of Guangxi and belong to rural areas. In 2009, the per capita net incomes of farmers in Rongxian county and Luchuan county were 4470 RMB and 4525 RMB, respectively (the average of Guangxi was 3980 RMB). In 2013, the per capita net incomes were 8135 RMB and 8342 RMB (the average of Guangxi was 7265 RMB). Three townships were selected in each county. Three villages were selected in each township, based on population size. Households were randomly selected from sample villages and all family members in a sampled household were individually interviewed.

### Data collection

The questionnaire was designed according to the purpose of investigating the progressivity of the health funding system and impoverishment due to medical expenses. The data mainly included sections on demographic data and expenditure data of households. Data on income tax rates and taxable household consumption were obtained from the China state administration of taxation (China Taxation Development Report: 2006–2010). Data on other payments for health care (out of pocket and social insurance) were obtained from survey responses. These two cross-sectional surveys were carried out in 2009 and 2013.

### Measuring progressivity

Vertical equity in healthcare financing is measured by analyzing the progressivity most commonly using the Kakwani Index of progressivity [6]. The Kakwani index is defined as twice the area between a payment concentration curve for a payment (for taxes or health care etc.) and the Lorenz curve for income (or other measure of ability to pay). The index’s value lies between −2 and 1. A negative index suggests regressivity (a lower proportion of income is paid out towards the payment as income increases) and a positive index suggests progressivity (a higher proportion of income is paid out towards the payment as income increases). The index, k, is calculated as

$$ \mathrm{k}=\mathrm{C}-\mathrm{G} $$

where C is the concentration index for the health-care payment and G is the Gini coefficient for the measure of ability to pay. The value of the index ranges from −2 to 1.

The overall progressivity of the health-care financing system can be determined by weighting the Kakwani index of each health-care payment identified at the household level based on the proportion that each payment makes up of total healthcare expenditure at national level [8].

### Concentration index

As a powerful and superior tool recommended by the World Health Organization to assess the degree of equity of health financing in different economic and social status [26], concentration index is defined as twice the area between the concentration curve and the line of perfect equality. The index’s value lies between −1 and 1. A negative value suggests the variable is concentrated in the poor, while a positive value suggests that the value is concentrated in the rich. Concentration curve is a graphical representation of the distribution of a variable of interest throughout the population with the population ranked by cumulative proportions from poorest to richest based on a living standard. If the particular variable is distributed proportionately through all the population, then the concentration curve is a diagonal line running at 45° from the origin (line of perfect equality).

### Gini coefficient

As Gini coefficient has been identified by the World Bank [26] as a robust indicator to evaluate the equity of health financing allocation, we used it to examine the fairness of health care financing. The summary measure associated with the Lorenz curve. Its value is twice the area between the Lorenz curve and the line of perfect equality. It has a value between 0 and 1 with zero indicating perfect equality. It is a commonly used measure of inequality in income distribution.

The Gini coefficient ranges from 0 to 1; higher Gini coefficient indicates greater inequities; a value of less than 0.2 suggests low inequities; a value of between 0.2 and 0.3 suggests moderate inequities; a value of between 0.3 and 0.4 suggests high inequities; a value of higher than 0.4 indicates extreme inequities.

### Catastrophic health expenditure

As a sensible indicator directly reflecting the burden of health payments in influencing household economy and causing impoverishment, we analyzed the incidence and intensity of catastrophic health spending. Catastrophic spending occurs when the ratio between total health expenditure and the difference between income and necessary expenditures such as accommodation, food, school, health care, clothing, water, electricity and sanitation exceeds 40% [27]. By calculating the incidence and intensity of catastrophic health expenditure, the effect of NCMS in financing risk was demonstrated.

### Variable definitions

The following definitions derived from the WHO document [28].

(1) Out-of-pocket health expenditure (oop).

Out-of-pocket health payments refer to the payments made by households at the point they receive health services [23]. Typically these include doctor’s consultation fees, purchases of medication and hospital bills. Although spending on alternative and/or traditional medicine is included in out-of-pocket payments, expenditure on health-related transportation and special nutrition are excluded. It is also important to note that out-of-pocket payments exclude any insurance reimbursement.

(2) Household consumption expenditure (exp).

Household consumption expenditure comprises both monetary and in-kind payment on all goods and services, and the money value of the consumption of home-made products.

(3) Food expenditure (food).

Household food expenditure is the amount spent on all foodstuffs by the household plus the value of a family’s own food production consumed within the household. However, it excludes expenditure on alcoholic beverages, tobacco, and food consumption outside the home (e.g. at hotels and restaurants).

(4) Poverty line (pl) and household subsistence spending (se).

The household subsistence spending is the minimum requirement to maintain basic life in a society. A poverty line is used in the analysis as subsistence spending.

There are many ways to define poverty. None of them are perfect considering the soundness in theory and feasibility in practice. Here we use a food share based poverty line for estimating household subsistence. This poverty line is defined as the food expenditure of the household whose food expenditure share of total household expenditure is at the 50th percentile in the country [25]. In order to minimize measurement error, we use the average food expenditures of households whose food expenditure share of total household expenditure is within the 45th and 55th percentiles of the total sample [25]. Considering the economy scale of household consumption, the household equivalence scale is used rather than actual household size. The equivalence scale is:

$$ \mathrm{eqsize}=\mathrm{hhsize}\ \upbeta $$

where hhsize is the household size. The value of the parameter β has been estimated from previous studies [23] based on household survey data from 59 countries, and is equal to 0.56.

(5) The household’s capacity to pay (ctp).

The household’s capacity to pay is defined as a household non-subsistence spending. However, some households may report food expenditure that is lower than subsistence spending (se > food). This indicates that the household’s food expenditure is less than the estimated poverty standard for that country. Such a situation could also be due to the fact that the reported food expenditure in the survey does not consider food subsidies, coupons, self-production and other non-cash means of food consumption. In this particular case the non-food expenditure is used as non-subsistence spending.

$$ \mathrm{ctp}=\exp -\mathrm{se}\ \mathrm{if}\ \mathrm{se}\le \mathrm{food} $$

$$ \mathrm{ctp}=\exp -\mathrm{food}\  \mathrm{if}\ \mathrm{se}>\mathrm{food} $$

(6) Catastrophic health expenditure (cata).

Catastrophic heath expenditure occurs when a household’s total out-of-pocket health payments equal or exceed 40% of the household’s capacity to pay or non-subsistence spending. The threshold of 40% can be changed according to countries’ specific situation [25].

The variable on catastrophic health expenditure is constructed as a dummy variable with value 1 indicating a household with catastrophic expenditure, and 0 without catastrophic expenditure.

$$ \mathrm{cata}=1\ \mathrm{if}\ \mathrm{oop}\ \mathrm{ctp}\ge 0.4 $$

$$ \mathrm{cata}=0\ \mathrm{if}\ \mathrm{oop}\ \mathrm{ctp}<0.4 $$

(7) Impoverishment (impoor).

A non-poor household is impoverished by health payments when it becomes poor after paying for health services.

The variable created to reflect poverty impact of health payments (impoor_h_) is defined as 1 when household expenditure is equal to or higher than subsistence spending but is lower than subsistence spending net of out-of-pocket health payments, and 0 otherwise.

$$ {\mathrm{impoor}}_{\mathrm{h}}=1\ \mathrm{if}\ {\mathrm{exp}}_{\mathrm{h}}\ge {\mathrm{se}}_{\mathrm{h}}\ \mathrm{and}\ {\mathrm{exp}}_{\mathrm{h}}-{\mathrm{oop}}_{\mathrm{h}}<{\mathrm{se}}_{\mathrm{h}},\mathrm{otherwise},{\mathrm{impoor}}_{\mathrm{h}}=0 $$

(8) Expenditure quintile (quintile).

The expenditure quintile is ranked by equivalized per capita household expenditure

$$ \left({\mathrm{eqexp}}_{\mathrm{h}}\right).{\mathrm{eqexp}}_{\mathrm{h}}={\mathrm{exp}}_{\mathrm{h}}\ {\mathrm{eqsize}}_{\mathrm{h}} $$

Note: household weight should be considered when grouping the population by quintile.

## Results

The numbers of households surveyed in 2009 and in 2013 were 4634 and 3951, respectively. The shares of total financing for the sources of health-care payments are summarized in Table 1.

**Table 1:** Shares of Total Financing

Quintiles of per capita consumption, gross	Per capita consumption, gross		Tax		Social insurance contributions		Out-of-pocket payments		Total payments		Per capita consumption, net of payments	
	2009	2013	2009	2013	2009	2013	2009	2013	2009	2013	2009	2013
Lowest quintile	4.4	6.5	5.0	6.4	18.9	17.4	8.7	9.7	11.3	11.5	1.7	4.9
2	9.1	11.7	9.7	11.6	18.5	17.7	13.3	18.0	14.1	16.1	6.9	10.3
3	14.6	16.7	14.6	17.0	20.6	20.1	14.5	20.8	16.7	19.5	13.3	15.8
4	23.8	22.9	23.4	22.6	21.5	21.0	20.8	24.0	21.8	22.5	24.5	23.0
Highest quintile	48.1	42.2	47.4	42.4	20.5	23.9	42.7	27.4	36.1	30.5	53.7	46.0
Total	100.0	100.0	100.0	100.0	100.0	100.0	100.0	100.0	100.0	100.0	100.0	100.0
Gini coefficient	0.4379	0.3546									0.5351	0.4130
(standard error)	(0.01)	(0.01)									(0.01)	(0.01)
Concentration index (standard error)			0.4247 (0.01)	0.3559 (0.01)	0.0298 (0.00)	0.0681 (0.00)	0.3360 (0.02)	0.1822 (0.01)	0.2515 (0.01)	0.1911 (0.00)		
Kakwani index			−0.0132	0.0013	−0.4081	−0.2865	−0.1019	−0.1724	−0.1864	−0.1636		
(standard error)			(0.00)	(0.00)	(0.01)	(0.01)	(0.02)	(0.01)	(0.01)	(0.00)		

The proportions of tax and out-of-pocket payments increased between 2009 and 2103 in the low and middle consumption quintiles and decreased in the high consumption quintiles. The total payments showed a similar trend. Social insurance contributions had a slight decline in all but the highest quintiles, which had a slight increase. Overall, the Gini coefficient of both per capita consumption gross and per capita consumption net of payments decreased from 0.4379 to 0.3576 and from 0.5351 to 0.4130, respectively. The concentration indices of tax decreased from 0.4247 to 0.3559 and for out-of-pocket payments decreased from 0.3360 to 0.1822 but there was an increase in social insurance. In 2013 the Kakwani index was negative in all indicators except for tax. However, the Kakwani index for tax, social insurance and out-of-pocket payments declined between 2009 and 2013.

**Fig. 1:**
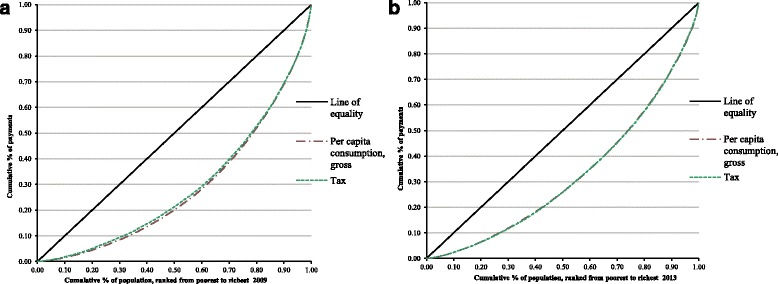
Concentration curves for health payments and taxes **a**. in 2009, **b**. in 2013

**Fig. 2:**
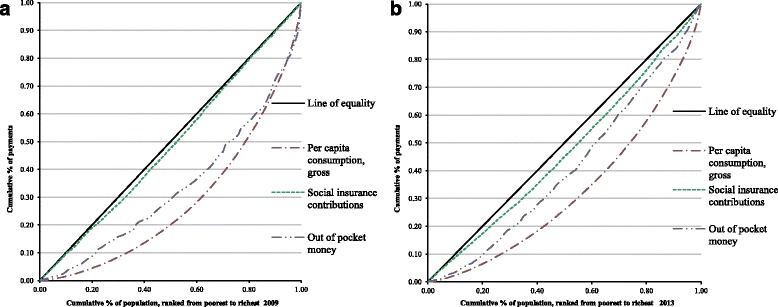
Concentration curves for health payments, insurance, and out-of-pocket payments: **a**. in 2009, **b**. in 2013

Figure 1a and b show concentration curves for health payments and tax in 2009 and 2013, respectively while Fig. 2a and b show concentration curves for health payments, social insurance contributions, and out-of-pocket payments in 2009 and 2013, respectively.

Table 2 presents a decomposition of the redistributive effect of the health care financing system for total consumption and the three health financing indicators. The three components: vertical effect (V), horizontal inequality (H), and reranking (R) are used to calculate V / RE, H / RE and R / RE which shows the proportions of inequity for each indicator.

**Table 2:** Decomposition of Redistributive Effect of Health Care Financing System

Quintiles of per capita consumption, gross	Per capita consumption, gross		Tax		Social insurance contributions		Out of pocket money		Total payments	
	2009	2013	2009	2013	2009	2013	2009	2013	2009	2013
Lowest quintile	4.4	6.5	5.0	6.4	18.9	17.4	8.7	9.7	11.3	11.5
2	9.1	11.7	9.7	11.6	18.5	17.7	13.3	18.0	14.1	16.1
3	14.6	16.7	14.6	17.0	20.6	20.1	14.5	20.8	16.7	19.5
4	23.8	22.9	23.4	22.6	21.5	21.0	20.8	24.0	21.8	22.5
Highest quintile	48.1	42.2	47.4	42.4	20.5	23.9	42.7	27.4	36.1	30.5
Total	100.0	100.0	100.0	100.0	100.0	100.0	100.0	100.0	100.0	100.0
Payments as fraction of Income (g)	1.0000	1.0000	0.0992	0.0697	0.1241	0.0872	0.1241	0.0872	0.3474	0.2441
Kakwani index assuming horizontal equity (Ke)	0.0000	0.0000	−0.0135	0.0013	−0.3416	−0.1988	−0.1067	−0.1706	−0.1000	−0.1359
Vertical effect (V)	0.4371	0.3535	−0.0015	0.0001	−0.0484	−0.0190	−0.0151	−0.0163	−0.0532	−0.0439
Horizontal inequality (H)	−0.0008	−0.0011	0.0001	0.0000	0.0107	0.0090	0.0024	0.0018	0.0519	0.0115
Reranking (R)	0.0000	0.0000	−0.0001	0.0000	−0.0157	−0.0038	−0.0055	0.0019	−0.0482	−0.0057
Total redistributive effect (RE = V - H - R)	0.4379	0.3546	−0.0015	0.0000	−0.0434	−0.0242	−0.0120	−0.0199	−0.0569	−0.0497
V / RE	0.9981	0.9968	0.9619	1.9321	1.1160	0.7857	1.2637	0.8175	0.9354	0.8835
H / RE	−0.0019	−0.0032	−0.0886	0.8764	−0.2472	−0.3704	−0.2002	−0.0882	−0.9113	−0.2311
R / RE	0.0000	0.0000	0.0505	0.0557	0.3632	0.1561	0.4638	−0.0943	0.8467	0.1146

In 2013 the vertical effect (V) of all the three financial indicators were negative except for tax. Some values of V exceeded 1, which indicates that vertical inequity has influenced total health financing inequity. The values for V / RE declined from 2009 to 2013, indicating that vertical inequity has improved in the five years. The values for H / RE did not change substantially over time, indicating that horizontal inequity had little effective improvement.

The incidence and intensity of catastrophic health payments are summarized in Tables 3, 4 and 5. The headcounts of catastrophic health payment had a sharp decline from 2009 to 2013, either using total expenditure (Table 3), which decreased from7.3% to 1.2%, or non-food expenditure (Table 4), which decreased from 26.1% to 7.5%. Although the concentrations indices of catastrophic health payment were negative, the absolute values increased (Table 5).

**Table 3:** Incidence and Intensity of Catastrophic Health Payments

	Threshold budget share					
	Headcount		Overshoot		Mean positive overshoot	
	2009	2013	2009	2013	2009	2013
Lowest quintile	12.3	2.7	8.5	0.1	69.1	3.7
2	10.5	2.0	3.9	0.1	36.7	4.5
3	4.0	1.1	0.3	0.0	7.6	4.4
4	3.9	0.3	0.3	0.0	6.9	5.9
Highest quintile	5.8	0.0	0.5	0.0	7.8	5.7
Total	7.3	1.2	2.7	0.1	36.6	4.2

**Table 4:** Incidence and Intensity of Catastrophic Health Payments, Using Non-food

	Threshold budget share					
	Headcount		Overshoot		Mean positive overshoot	
	2009	2013	2009	2013	2009	2013
Lowest quintile	45.6	11.1	28.9	0.6	63.4	5.5
2	28.6	14.6	14.1	0.8	49.4	5.7
3	20.4	5.4	4.6	0.4	22.6	7.5
4	18.9	4.7	5.0	0.3	26.6	6.6
Highest quintile	17.2	1.7	4.8	0.1	28.0	6.3
Total	26.1	7.5	11.5	0.5	44.0	6.0

**Table 5:** Distribution-sensitive Catastrophic Payments Measures

	Threshold budget share:40%			
	Using total expenditure		Using Non-food	
	2009	2013	2009	2013
Concentration index, C_E	−0.216	−0.477	−0.207	−0.326
standard error	0.04	0.05	0.02	0.03
Concentration index, C_O	−0.659	−0.451	−0.433	−0.300
standard error	0.04	0.06	0.03	0.04

Table 6 shows a comparison of measures of poverty based on gross consumption and net spending on health care in 2009 and 2013. Poverty increased over time for both measures. However, the normalized poverty gap decreased for net spending and the normalized mean positive poverty gap decreased for both measures.

**Table 6:** Measures of Poverty Based on Gross Consumption And Net of Spending on Health Care

	Gross consumption		Net spending	
	2009	2013	2009	2013
Poverty headcount	13.0	15.4	18.4	22.5
Poverty gap	52.0	110.5	106.7	158.7
Normalized poverty gap	4.3	4.8	8.9	6.9
Normalized mean positive poverty gap	33.3	31.3	48.4	30.6

Poverty line in 2009 and 2013: 1196.0 RMB and 2300.0 RMB, respectively

**Fig. 3:**
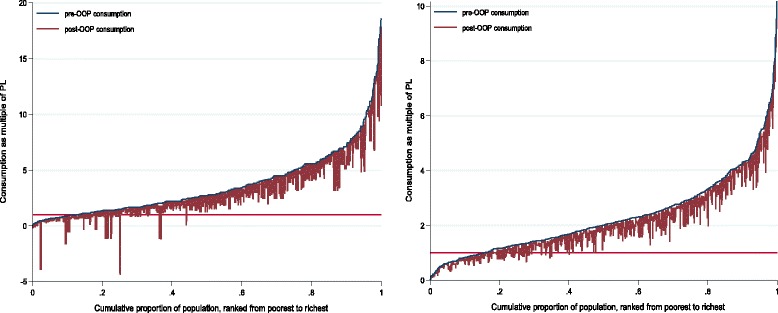
Effect of Health Payments on Household Consumption: 2009 (left panel) and 2013 (right panel)

Figure 3 compares household consumption in 2009 (left) and 2013 (right) using Pen’s Parade graphs. The x-axis is the cumulative population percentage per capita household consumption. The y-axis is the multiple of the poverty line, and PL is the poverty line. Overlaid on the chart are the out- of-pocket payments of each household. Catastrophic health care payments decreased in 2013 as seen by the intersection of the polyline and PL moving to right in the x-axis.

## Discussion

Our results demonstrate that there is a significant potential to improve the financial protection of rural Guangxi population through the expansion of NCMS from 2009 to 2013. In these five years, NCMS has raised its premium from 100 RMB to 340 RMB per person, and broaden its reimbursement scope both in inpatient and outpatient services. The dropping down of catastrophic health payments headcounts and the share of OOP in total payment provides evidence to the effectiveness of financial risk pooling intervention by NCMS, and this approach indeed help reduced the financial barriers to health care services.

The healthcare financing systems in Luchuan and Rongxian counties were found to be regressive. It was likely that the regressive nature of social insurance payments was the chief contributor to this since, even though all of the payments were disproportional, the absolute values of the Kakwani index for social insurance were bigger than for other payments (Table 1). The uneven distribution of wealth has thus eased in some way. The results showed that the funding system was regressive, and the number of health services financing the study participants was tired, that is, the amount and proportion of health services that were assumed by different groups decreased with increasing consumption. From Fig. 1a and b, it’s clearly seen that the concentration curves of health payments and tax almost coincide, but the curves in 2013 come closer to the equality line than 2009,which show that tax become more equal as a health funding source. And this trend shows more obvious in Fig. 2a and b, both social insurance and out of pocket payment have improved in equality from 2009 and 2013. To a certain extent, this indicates that the policy of NCMS, which aims to relieve the burden of medical payments among people living in rural areas, did not meet expectations well. And about the role of tax in promoting progressivity, it didn’t show much help as it was clearly seen that the proportions of tax were lower than social insurance and out of pocket payment (Table 2). Nonetheless, the incidence of catastrophic health expenditures declined in 2013, as did the poverty headcount, using total expenditure from 7.3% to 1.2%, and using non-food expenditure from 26.1% to 7.5%. The difficulty here is that the poverty line for pre-payment income ought to include an element for health spending, whilst the poverty line for post-payment income ought not. This means that whilst some people may not be poor before health spending and be poor after it, there will be some who are marginally poor before health spending but not poor after it (they spend nothing on health care or they spend appreciably less than the health spending component of the pre-payment poverty line). Thus, whereas in the case where the extreme poverty line is used poverty will necessarily be higher ‘after’ health spending than ‘before’, in the case where the poverty line covers food and non- food items, poverty may, in fact, be higher pre- payment than post-payment. However, this encouraging change is likely due to the improved coverage of NCMS, which increased from 86.3% in 2009 to 98.1% in 2013. Funding premiums also had a strong enhancement from 100 RMB per person to 340 RMB per person.

The Gini coefficient for per capita consumption decreased from 0.4379 to 0.3546 (gross), and from 0.5351 to 0.4130 (net), respectively, which implied that with the passage of time and the implementation of new policies (especially NCMS and new medical reform), the extent of unfair wealthy distribution between populations in different socio-economic levels has eased. The concentration indices were all positive, which shows a higher concentration of persons with higher levels of payments in the wealthier quintiles. Nevertheless, a decrease in the concentration index for all the three health financing indicators indicates that health financing equity is improving.

The incidence and intensity of catastrophic health payment due to health expenditure decreased significantly. By comparing the headcounts and poverty gaps before and after out of pocket spending in Fig. 3, one can get a sense of its impoverishing effects, whether in terms of additions to the number of people classified as extremely poor or in terms of deepening poverty amongst the extreme poor [24]. Out-of-pocket payments have an unneglected impact on the headcount in the case of the broader-based poverty line. In addition, the normalized mean positive poverty gap narrowed (Table 6), which indicates that the government has implemented some effective policies to improve people’s livelihood.

As we mention in the introduction, China has begun the health insurance scheme in rural areas in 2003. However, the scheme is being regarded as a catastrophic health payment program in the past which only covers hospital payments. Since the hospitalization rate is relatively low and the inconvenient geographic transportation, very few peasants can benefit from this program directly due to low outpatient visits. It is reasonable to deduce that the new medical reform and the expansion of NCMS coverage to include outpatient services as well as hospital services has maximize the impact on health payment. What’s more, the results from our subgroup analysis also meet our expectation. We expected that NCMS would have a larger effect on low-economic groups than high-economic groups, and the extent of impoverishment headcount decline (Tables 3 & 4) can prove this hypothesis.

Compared with the study on out-of-pocket payments in Shandong Province [29], the proportion of urban residents who had out-of-pocket payments over the household non-food consumption increased from 7.4% to 9.0%, while for rural residents it increased from 10.0% to 12.0%. Thus, the level of medical expenses for residents is increasing, with rural resident paying a relatively higher amount, and a high burden for low income households. Shandong is a rich and developed province in China. High income families tend to spend more for health management and prevention, which is still a low proportion of their income. However, for poor families, who cannot afford health insurance out-of-pocket health payments are high. In Guangxi, after universal coverage, out-of-pocket payments have decreased from 12% to 8%, providing a successful experience to the less developed province.

Compared with surveys from other similar cities under comprehensive community health reforms conducted in different areas of China [30], the redistributive effect (RE) of family tax financing in eastern, central, and western areas were negative, (−0.0131,-0.0032 and −0.0012, respectively). In the eastern area, social medical insurance under health financing redistribution (RE = 0.0028) was higher than the west (RE = −0.0004) and the central area (RE = −0.0016. The RE for out of pocket expenditure in the east, middle and west was −0.0088, −0.0091 and −0.0055, respectively. This shows that urban employees’ medical insurance played a weak positive role in health financing redistribution; tax, urban residents’ medical insurance and out-of-pocket payments did not play a positive role in redistribution.

Compared with Thailand, another developing country in Asia well known for its success in health financing equity, our study showed a similar trend after universal health insurance coverage. Before 2002, the three existing basic medical security systems of Thailand covered 30% of the population, and the surplus population participated in the universal coverage (UC) policy of medical security system which promulgated in 2002 [31, 32]. Since the implement of the UC policy, it has improved the equity of medical service application and health financing, and reduced catastrophic health payments, and the spending burden of catastrophic health expenditure in low-income groups decreased from 6.11 (2000) to 4.65 (2002). In China, after 5 years of universal coverage, improvements have been noticeable, with headcounts of catastrophic health payments decreasing from 7.3% (2009) to 1.2% (2013), which shows that the policy has been effective for relieving the health care burden for peasants.

Compared with a study in Tianjin [33], a rich city in China, the Kakwani indices were respectively 0.0177 (tax), 0.0025 (social insurance), and 0.1479 (out-of-pocket payments), while the redistributive effects were −0.0003 (tax), −0.0006 (social insurance), and 0.00002 (out-of-pocket payments). The V/RE, H/RE, R/RE values for tax were −92.8%, 7.2% and 0.001%, respectively and the V/RE, H/RE, R/RE for out-of-pocket payments were 106%, 6% and 0.003%, respectively. In this case, the poor afforded more in tax, and the progressivity of social insurance has been impaired by horizontal inequity.

In another study from Xinjiang autonomous region in far northwestern China [34], a similar area as Guangxi in terms of economic level, the concentration index for tax expenditure was 0.33 in 2003 and 0.36 in 2008, and the Kakwani indexes were −0.05 and −0.02, respectively, indicating that tax financing tended to equal proportion. The concentration indexes for social health expenditure in 2003 and 2008 were −0.33 and 0.15, and the Kakwani indexes were −0.71 and −0.23, respectively. The concentration indexes for commercial health insurance expenditure in 2003 and 2008 were 0.64 and 0.58, and the Kakwani indexes were 0.25 and 0.20, respectively. The concentration indexes for out-of-pocket payments in 2003 and 2008 were 0.43 and 0.50, and the Kakwani indexes were 0.04 and 0.12, respectively. All these results indicate that the health finance by tax changed little relative to a person’s ability to pay, and the NCMS improved the equity of social health security expenditure in rural areas, commercial health insurance increased for poor families, and out-of-pocket payments among wealthy families increased. In summary, results from Xinjiang are consistent with our study.

In summary, according to the empirical findings conducted in other studies, we concluded that in developed provinces, both urban and suburban social insurance failed to performe significantly better in reducing regressiveness of health funding system and easing health financial burden, while in developing districts or rural areas, NCMS showed vibrant and remarkable progress which can support the conclusion in our research. Based on these comparison, rural areas should insist on carrying out NCMS and the government should improve the concreate policies aiming to narrow the gap between urban and suburban, especially enhancing the tax regulation in the whole scheme, and the introduction of progressive mechanism in NCMS system should be considered.

NCMS is a highly subsidized health insurance scheme, and government financing comprised more than half of the annual NCMS pooling revenue. For example, central and local government financing in 2009 accounted for 28.55% and 49.98% of NCMS revenue, respectively [35]. According to the NCMS setting, which allows provinces to have some freedom in certain areas, such as how to design subsidy policies for lower-income people, the choice of tax rates, and the choice of how much of the total expenditure has to be financed through taxation and how much through mandatory health insurance. In addition, the NCMS officials might tend to set the deductibles too high and the copayment rates too low in order to guard against bankruptcy of the NCMS system if an unexpected accident happened such as widespread disease outbreak. Therefore, in our case, as a minority and poor region, it is crucial for Guangxi to transform this given amount of spending on health into effective and efficient health services so as to meet the two universal rural health insurance goals of helping rural residents minimize catastrophic financial risks and improve their health status.

Firstly, payment regulation reform based on clinical pathway is an alternative to control unnecessary medical cost and improve the health service quality. There is anecdotal evidence that some hospitals use more costly procedures or equipment for NCMS participants in response to the implementation of the NCMS [36]. So participants’ relative out-of-pocket expenses may not necessarily be lower than before NCMS participation. So pilot reform for the provider payment method has been tried in some areas such as DRGs-PPS (Diagnosis Related Groups-Prospective Payment System). This resulted in a positive change in health provider behaviors and reduced out-of-pocket payments for patients [37]. And along with the introduction and development of DRGs-PPS, the information systems construction including electronic medical records in health facilities and its interlinkage across health facilities should be enhanced in rural areas.

Secondly, NCMS benefit package should fit into the disease profile and health expenditure pattern of the population [38]. Chronic diseases, needing not only hospitalization service but also ambulatory care and drugs, are responsible for 68.8% of the total disease burden in China. What’s more, the epidemic of chronic diseases, is becoming the main cause of catastrophic health expenditure, will rise in the future [39]. So,adding some common but costly chronic diseases to the NCMS reimbursement list can help eased the health financial risk of residents.

Lastly, in the province which has a great proportion of agricultural population as Guangxi, NCMS is an essential tool to provide health financial protection for residents, but it is still not enough. Maybe the powerful introduction of different commercial insurances can be a promising way to improve health financial fairness.

From the results of our study, the equity of health financing in rural areas of China still needs to be improved,at least in Guangxi’s case.

The results contained in this work shed light on some aspects that should be considered in other undeveloped areas especially rural areas that are planning to use the regulation of NCMS and premium subsidies to ensure universal coverage. Although the combination of NCMS and premium subsidies might be in theory the best solution in terms of (vertical) equity, in the daily practice of different provinces the empirical evidence can point to a different outcome. And the promotion of NCMS indeed help alleviate the health financing inequity and protect peasants avoid from catastrophic health payment.

Additionally, the utilization of the data to represent a single whole may has its limitation. However, we carefully selected our sample sites and calculated the sample size, tried our best to make the data reflect the actual situation in the most of the rural areas according to our purpose. Our study may not cover all the information in rural Guangxi, but in the average level,we can see it as reliable.

### Limitations

Our study has some limitations which should be acknowledged. Self-reported responses used in this investigation is a common way to evaluate the level of personal income and health expenditure. Reporting bias is a major disadvantage in the assessment of economic levels because of self-reported data. This study compared the changes of health financing equity in target areas, and analyzed and imposed some policy recommendations on the basis of results. However, the quantitative analysis of the factors affecting the health equity is difficult to implement due to the small number of data samples, but it can be supplemented in the follow-up study. Finally,our study only compared health financial inequity between different economic groups. No outpatient and inpatient health expenditures comparison was included, so we are not able to figure out more impacts in NCMS implementation. In spite of these limitations, our study still provides useful policy information on the development of NCMS in developing countries, and also identifies areas where further research is needed.

## Conclusion

After the introduction of the new health care reform in Yulin and Rongxian counties, health inequity has improved as the universal health coverage broadening, the incidence of catastrophic payments has decreased, and the poverty gap is narrowing, which indicates that China’s new medical reform especially NCMS has had some effect in Guangxi. However, health funding fairness still has some challenges, more attention should be drawn on lowering the out of pocket payments and improving the tax financing mechanism.

## Abbreviations

NCMS: New Cooperative Medical Scheme
OOP: Out Of Pocket Payment
